# Authority and Responsibility

**Published:** 1905-08-19

**Authors:** 


### The Hospital.

A Journal of

The Medical Sciences and Hospital Administration.

Vol. XXXVIII.—No. 986 SATURDAY, AUGUST 19, 1905.

### Authority and Responsibility.

It seems to be almost a self-evident proposition, and is yet one by no means generally acted upon, that persons placed in positions of authority by the suffrages of their fellow-citizens are morally bound, and ought also to be bound legally, to exercise the powers conferred upon them for the general good of the community. We had to comment, last week, upon the fact that certain metropolitan boards of guardians habitually fail to avail themselves of the resources provided, by the Metropolitan Asylums Board, for the better care and treatment of several classes of specially afflicted children, and that, in consequence, not only are these children comparatively neglected, but the ratepayers, whose interests it is also the duty of the guardians to protect, are practically called upon to pay twice over for their maintenance, besides paying for the special advantages which the children do not receive. The conditions upon which light was thrown by the report of the Asylums Board are more or less repeated, in one form or another, all over the kingdom; insomuch that it would certainly be difficult, and would probably be impossible, to find a single local authority really bent upon doing the very best that is possible for the community placed under its care. In every, or almost every, rural district in England and Wales, and in the majority of urban districts as well, maladministration or non-administration of sanitary law is the rule rather than the exception. When the authorities of a big town deliberately prepare a population susceptible of small-pox, or deliberately neglect the provision of a proper water supply, and are rewarded by an epidemic of typhoid fever, the whole affair is sufficiently talked about to become a nine days' wonder; but similar conditions on a smaller scale, although they are almost universal, yet attract little or no notice. Wherever the Medical Inspectors of the Local Government Board go, they have, as a rule, the same tale to tell. Insanitary dwellings, imperfect removal of filth, and defective water supply are the conditions found in varying combinations and in varying degrees of intensity, almost everywhere, until the very monotony of the descriptions of them goes far to deprive them of interest. The laws practically provide no remedy; and the prevailing neglect rests upon a solid foundation of municipal ignorance, indifference, and parsimony, against which the forces of individual knowledge and enlightenment are condemned to strive in vain.

It is an interesting question of casuistry whether right-minded persons are entitled to derive satisfaction from the discovery that others are at least as badly off as themselves; but, if this question can be answered in the affirmative, we in this country might, perhaps, be justified in turning our eyes towards the United States in search of consolation for our own sanitary and administrative deficiencies. The great city of New Orleans is being ravaged by yellow fever, just as Lincoln has lately been ravaged by typhoid, and as Derby, in all probability, will before long be ravaged by small-pox. At the time of writing these words, the cases in New Orleans have exceeded a thousand in number, and the deaths have been at the rate of about one in six of the cases. Eminent persons, among them the Archbishop of the diocese, have been struck down. The meaning of this is that, even with the example of Havana before their eyes, the municipal authorities of New Orleans have neglected to avail themselves of the simple and efficient means of protection which science has offered to them, and that they are suffering the natural consequences of their neglect. A considerable time has now passed away since a devoted band of American army surgeons, and of volunteer subjects for experiment, who had faith in the conclusions of science, established beyond the reach of doubt that yellow fever is not contagious, and that the sole channel of its conveyance from man to man is by the bites of a mosquito of the genus *Stegomyia*, which thrives only under the conditions of climate and temperature which have hitherto determined the geographical limitations of the disease. In Havana, where yellow fever has usually shown itself every year, and where it has frequently occasioned great mortality, it may perhaps be assumed that the recent war has left its traces in the form of a system of local government which has some sense of its responsibilities towards the governed; and the new knowledge was at once brought into fruitful application. The larvæ of *Stegomyia* and their lurking-places were sought for much as the larvæ of *Anopheles* and their lurking-places have been sought for in Ismailia and in other localities in which the researches of Professor Ronald Ross and his fellow-workers have been suffered to bear fruit; and the result has been that Havana, we believe for the first time in its recorded history, has passed through one mosquito season without a single recorded case of the disease. New Orleans will, we presume, be equally wise after the event.

The acceptance by the Mayor of the offer of the Liverpool School of Tropical Medicine to send Professors Ronald Ross and Boyce to the assistance of the municipality appears to show that the gentlemen composing this body are not too proud to learn; and, if this be the case, it may be predicted with some confidence that the epidemic of yellow fever from which the city is now suffering will be the last that it is likely to experience. It is much to be wished that the same hope could be entertained concerning the multitude of minor and comparatively inconspicuous epidemics of various kinds which every year make up an appalling total amongst ourselves. The health of the lowest classes of our people is to an enormous extent being thrown away by municipal incompetence and negligence; and we are calling out to Hercules when we ought to put our own shoulders to the wheel. We do not want philanthropic associations half so much as we want laws which mayors and town councillors shall themselves be compelled to obey, under pain of penalties for failure or for recalcitrancy.

